# Academic and Clinical Nurses' Perceptions and Experiences on Academic-Practice Partnership in Evidence-Based Practice: An Interpretive Description

**DOI:** 10.1155/2023/2955731

**Published:** 2023-09-19

**Authors:** Qirong Chen, Xirongguli Halili, Wenjun Chen, Junqiang Zhao, Aimee R. Castro, Siyuan Tang, Honghong Wang, Yuting Xia, Guiyun Wang, Chongmei Huang

**Affiliations:** ^1^Xiangya School of Nursing, Central South University, Changsha 410000, China; ^2^Xiangya Center for Evidence-Based Practice & Healthcare Innovation: A Joanna Briggs Institute Affiliated Group, Central South University, Changsha 410000, China; ^3^School of Nursing, Faculty of Health Sciences, University of Ottawa, Ottawa K1N 6N5, Canada; ^4^Waypoint Research Institute, Waypoint Centre for Mental HealthCare, Penetanguishene, Ontario, Canada; ^5^Dalla Lana School of Public Health, University of Toronto, Toronto, Ontario, Canada; ^6^Ingram School of Nursing, McGill University, Montreal H3A 2M7, Canada; ^7^School of Nursing, Shandong Xiehe University, Jinan 250000, China

## Abstract

**Aim:**

To explore academic and clinical nurses' perceptions and experiences on academic-practice partnership in evidence-based practice.

**Background:**

Academic-practice partnership could promote evidence-based practice which is crucial for high-quality care. Academic and clinical nurses are the foundation of the partnerships; however, there is little knowledge of their perceptions and experiences on academic-practice partnership in evidence-based practice.

**Methods:**

This is an interpretive description study. Twenty-two eligible participants were interviewed through face-to-face or online videoconferencing meetings. Guiding questions for the interviews focused on the perceptions and experiences of academic-practice partnership in the context of evidence-based practice. Interviews were recorded, transcribed, and checked verbatim. We used constant comparative analysis to analyze the qualitative data.

**Results:**

Four themes with fifteen subthemes were generated: necessities, modes, challenges, and benefits of the academic-practice partnership in evidence-based practice. Participants believed that academic-practice partnership was a necessary strategy to promote evidence-based practice and could be built through different modes. Nevertheless, most academic-practice partnerships were superficial because of specific types of challenges. However, a good academic-practice partnership could create a win-win situation for both nursing academia and clinical practice.

**Conclusion:**

Academic-practice partnership is a win-win strategy for both the academic side and clinical side to promote evidence-based practice. Different modes of academic-practice partnership provide academic and clinical nurses with more opportunities to promote evidence-based practice with a higher likelihood of successful implementation. However, related challenges require multilevel measures to provide better environments to initiate, build, and maintain intensive collaborations between academic and clinical nurses. *Implications for Nursing Management*. Academic and clinical organizations, leaders, and individuals should take multilevel measures to initiate, build, and maintain a close academic-practice partnership to promote evidence-based practice, which is crucial for high-quality nursing care, patient safety, and nursing discipline development.

## 1. Introduction

Evidence-based practice (EBP) in nursing involves using the best available evidence to make nursing care decisions, by combining nurses' clinical expertise, the client's values and preferences, and the available resources [1]. EBP has been recognized as an effective way for nurses to provide patients with high-quality and safe nursing care [2]. It is also crucial for promoting the development of nursing as a profession and a discipline [3, 4]. The benefits of EBP have been recognized and appreciated by many healthcare policymakers, nursing executives, clinical nurses, and academic nurses [3, 5]. The foundations for EBP in nursing (e.g., nursing evidence based on high-quality studies, theoretical frameworks for EBP, and education resources) have been developed for decades [5, 6]. However, there are many barriers to EBP, leading to an unfortunate lack of EBP in most clinical settings, especially in low- and middle-income countries [7].

A systematic review of EBP in low- and middle-income countries found that there were multilevel barriers to implementing and sustaining EBP: institutional barriers, intradisciplinary barriers, and individual nurse-related barriers [7]. Institutional barriers to EBP included scant resources, limited access to information, inadequate staffing, and a lack of institutional support. Intradisciplinary barriers included a lack of communication between academic and clinical nurses, inconsistencies between education and practice in the nursing discipline, and lack of teamwork. Individual nurse-related barriers included the individual clinician's lack of time, high workload, inadequate knowledge and skills, and resistance to changes. However, among these barriers, lack of resources, insufficient evidence-based practice knowledge and skills, and low English proficiency of clinical nurses (in non-English-speaking countries) are the key barriers requiring solutions [1, 4, 8, 9]. Addressing these key barriers is the foundation for further providing institutional resources to promote EBP; academic-practice partnership offers a critical strategy for overcoming these barriers.

Academic-practice partnership has been recognized as having great potential to promote the implementation of EBP in nursing [10–12]. Academic-practice partnership is a formal or informal arrangement between cooperating parties to advance mutual interests [13]. The American Association of Colleges of Nursing (AACN) suggested that “academic-practice partnerships are an important mechanism to strengthen nursing practice and help nurses become well positioned to lead change and advance health” [14]. Researchers have suggested that academic-practice partnership may address the key barriers to EBP (i.e., lack of resources, insufficient knowledge and skills, and low English proficiency) by sharing resources across academic and clinical settings [10–12].

Based on a systematic and comprehensive literature search, we found that the existing evidence on academic-practice partnership in EBP was limited. Only 26 case reports were identified as related evidence in the systematic literature search and screening [15]. Furthermore, most literature discussed academic-practice partnership on an institutional level with a macro perspective, with limited focus on the individual perspectives of academics and clinicians regarding academic-practice partnership [10, 13, 16].

Yet, individual academic nurses (i.e., academic staff and postgraduate students in university nursing departments) and clinical nurses (i.e., head nurses and bedside nurses in hospitals) form the foundation and core of academic-practice partnerships because they are the persons involved in the specific collaboration activities [11]. A recent quantitative study indicated that academic and clinical nurses all have a high demand for collaboration in EBP [17]. Therefore, more individual-level studies focusing on academic and clinical nurses are necessary for the promotion of academic-practice partnership in EBP. To promote individual-level studies, it is essential to have a comprehensive understanding of the perceptions and experiences of academic and clinical nurses on academic-practice partnership in EBP. Such evidence is crucial for the development of related theoretical frameworks, models, and interventions for promoting academic-practice partnership in EBP. However, there is a lack of individual-level evidence on specific perceptions and experiences of academic and clinical nurses on academic-practice partnership in EBP.

## 2. Methods

### 2.1. Aim

This study aimed to explore academic and clinical nurses' perceptions and experiences of academic-practice partnership in EBP.

### 2.2. Design

This is an interpretive description study based on the interpretivist paradigm [18]. This methodology could orient data analysis towards the development of findings that contribute to the final goal of our project, which is to develop a theoretical model and an intervention for academic-practice partnership in EBP [18, 19]. This study was reported following the COREQ checklist.

### 2.3. Participants

In this study, we defined “*academic nurses*” as academic staff or postgraduates in university nursing departments and “*clinical nurses*” as head nurses and bedside nurses working in hospitals.

Inclusion criteria for academic nurses were as follows: (1) nursing academic staff were responsible for teaching and/or research in nursing schools or universities and had completed a systematic EBP training program(s); (2) postgraduate nursing students were master or doctoral students who had completed an EBP course in a university nursing department. Inclusion criteria for clinical nurses were as follows: (1) head nurses were responsible for managing at least one unit in a hospital setting and had received EBP-related training. (2) Bedside nurses were responsible for providing direct care to patients in a hospital unit and have received EBP-related training. The inclusion of these four types of nurses was to provide diverse perspectives from both academic and clinical nurses, who are the most involved stakeholders in the academic-practice partnerships.

Participants were recruited through purposive and snowball procedures to improve the representativeness of the study sample [20]. One clinical nurse declined to participate in the interview due to heavy workload. 22 interviews were conducted to reach data saturation. General information about the participants is shown in Table 1 and the Supplementary Material Table S1, which was helpful for readers to understand the quotes cited in the results.

### 2.4. Data Collection

The data were collected between November 2021 and March 2022. Individual semistructured interviews were conducted through online videoconferencing meetings or face-to-face meetings in hospitals or in the office of the interviewer. The duration of the interviews ranged from 23 to 63 minutes.

The interviews were conducted by the first author (Q.C., Ph.D.) who has prior experience in conducting qualitative studies and is a core member of a JBI Affiliated Group for promoting EBP in a School of Nursing. Before the formal data collection, the interview questions were piloted in two interviews, and no adjustments were required. Therefore, the data collected from these piloted interviews were also included in the data analysis. The following key questions guided the interviews: (1) How do you think about academic-practice partnership in EBP? (2) Could you describe your experience of academic-practice partnership in EBP? (3) Will you participate, or why have you participated, in an academic-practice partnership for EBP?

Interviews were recorded, transcribed, and checked verbatim. Furthermore, a self-designed questionnaire was used to collect demographic information and some general information related to EBP (e.g., training experience and experience in academic-practice partnership) of participants. EBP competencies of participants were also measured by the Evidence-Based Nursing Practice Competence Scale which has good reliability and validity [21].

### 2.5. Data Analysis

The 22 interview transcriptions were uploaded and analyzed in NVivo 12.0. After transcribing the data, the first author (Q.C.) worked closely and intensively with the texts to conduct inductive coding for insights into the participants' experiences and perspectives. The second author, a Ph.D. candidate who has completed the qualitative study course and has experience in conducting qualitative studies, checked the coding of the texts. The constant comparative analysis involves the following six steps: immersing in the data, developing an initial thematic template, organizing the data based on the template, condensing data and reflecting, comparing and contrasting data within similar participant categories, and comparing and contrasting data with different participant categories [19, 22]. The identification of themes and subthemes evolved as both authors had multiple discussions. Four of the participants (one nursing academic staff, one nursing postgraduate student, one head nurse, and one bedside nurse) were invited to discuss and verify the results of the data analysis.

### 2.6. Rigour

The following strategies were used to improve the trustworthiness and rigour of this study [19, 23]. (1) We thoroughly immersed ourselves in collecting, analyzing, and understanding data, in order to ensure the saturation of data. Furthermore, data analysis was performed along with the data collection. (2) Participants had the freedom to speak, participants' voices were fully heard, and participants' perceptions were accurately represented. (3) Considering the role of promoting EBP and academic-practice partnership, the primary researcher may hold an inclination towards academic-practice partnership in EBP. Therefore, the primary researcher used a reflexive diary to maintain reflexivity and delimit subjectivity. (4) Member checking: member checking is a qualitative technique used to establish the tenet of credibility in trustworthiness. Two researchers were responsible for data analysis. Considering the time, cost, efficiency, and effectiveness comprehensively, we invited four of the participants to individually check our results to confirm the interpretation. (5) Thick and contextualized description: we provided specific information about the participants, which could help readers to understand the context of this study better. (6) Researcher credibility: the researchers either have Ph.D. degree or are Ph.D. candidates and all of them have completed systematic training in qualitative research.

### 2.7. Ethical Considerations

This study was approved by the Institutional Review Board of Xiangya School of Nursing, Central South University (No. E2021130). Informed consent was obtained from all participants. Each participant was informed of the goal and methods of this study, received a guarantee of confidentiality and anonymity, and agreed that the interview would be recorded. Only research group members could access the data, and potential identifiers of participants were removed from the quotes in the results.

## 3. Results

Four themes with fifteen subthemes relating to academic-practice partnership in EBP (Table 2) were generated: necessities, modes, challenges, and benefits of the academic-practice partnership. Participants believed that academic-practice partnership is a necessary strategy to promote EBP, and such partnerships could be built through different modes. Nevertheless, most current academic-practice partnerships are superficial and tokenistic because of common and specific challenges. A good academic-practice partnership could ideally create a win-win situation for both nursing academics and clinicians.

### 3.1. Necessities of Academic-Practice Partnership in Promoting EBP

All participants considered academic-practice partnership to be an indispensable for implementing and sustaining EBP. The complementary competencies and resources of the academic and clinical roles can offset the limitations of each other to promote EBP.

#### 3.1.1. Complementary Competencies for Partnership

All participants described that academic and clinical nurses had necessary and complementary competencies for conducting the key steps of EBP (i.e., proposing clinical problems, generating structured questions for evidence search, searching for evidence, appraising evidence, synthesizing evidence, disseminating evidence, and implementing evidence). All participants acknowledged that *“Academic nurses have more theoretical and methodological knowledge related to evidence-based practice. … Clinical nurses have more clinical expertise, experience, and context knowledge.” (Participant 10, Ph.D. student)*

Considering that EBP is a combination of theory and practice, the complementary academic and clinical competencies of academic and clinical nurses are necessary for EBP. Academic nurses focus more on the steps requiring theoretical and methodological knowledge and thus are more competent in working with literature (i.e., evidence search, appraisal, and synthesis). As stated by one participant, *“Overall, in evidence search, appraisal, and synthesis, academic nurses have significantly better competencies than clinical nurses.” (Participant 6, academic staff)* However, clinical nurses have lived experience in dealing with complex and emergent clinical problems and thus offer valuable insights into proposing clinical problems for further research. For example, *“Clinical nurses could propose many current and real-world clinical problems which are urgent to be solved. These problems are often specific and the starting points of evidence implementation projects. For academic nurses who do not have current clinical knowledge and experience, and do not know organizational environments and culture, it is impossible to propose these clinical questions.” (Participant 4, academic staff)*

For other steps of EBP (i.e., generating structured questions for evidence search, evidence dissemination, and implementation), academic and clinical nurses play complementary roles, rather than one role being more competent in these specific steps. For example, one participant suggested that *“Academic nurses could better frame clinical questions into structured questions for evidence-based practice projects.” (Participant 10, Ph.D. student) Another participant shared, “If the PICO [EBNP question] is related to clinical practice, it may be addressing issues that I have clinical blindspots or limited knowledge of. I will need help from clinical nurses to determine the specific inclusion and exclusion criteria of PICO.” (Participant 12, master student)* For evidence dissemination, participants mentioned *“Academic nurses have a better competence in academic writing and presentations, which is essential for disseminating evidence through academic journals or conferences. … Clinical professionals have a better competence in patient education and communication, which is essential for disseminating evidence to patients and relatives in the clinical practice context.” (Participant 19, clinical nurse)* For evidence implementation, participants described that *“Clinical nurses always know the crux of the matter better. They know the feasibility of the evidence implementation plan better.” (Participant 8, Ph.D. student) “Academic nurses could provide us with some suggestions related to theoretical frameworks and methodology when we are designing the evidence implementation projects.” (Participant 21, clinical nurse)*

#### 3.1.2. Complementary Resources for Partnership

Most clinical participants expressed that they have insufficient resources for evidence synthesis. *“When I did the evidence searching,… I was not confident whether my literature search was comprehensive because I do not have the access to many databases. This limited me a lot. … If we were in an academic-practice partnership, we hope we could get database support from universities.” (Participant 22, clinical nurse)* The academic-practice partnership could offset the limited resources for evidence synthesis on most clinical sides.

For evidence dissemination, the academic side and clinical side have different resources (e.g., human resources, media resources, and available targeted populations). *“For evidence dissemination, academic organizations have better resources for disseminating evidence through academic publications, conferences, and academic media mainly targeted on healthcare professionals and academics, while clinical organizations mainly disseminate evidence through clinical professionals, booklets, and patient groups mainly targeted on clinical nurses, patients, and relatives in the hospital.” (Participant 17, head nurse)*

Clinical organizations had exclusive resources for evidence implementations, such as clinical nurses, patients, head nurses with leadership, and clinical practice environments. *“Clinical side has its unique advantage-it is both the starting point and ending point of evidence because we need to first generate and finally use evidence in the clinical context.” (Participant 6, academic staff) “The clinical side has evidence recipients, evidence implementers, and clinical practice environment for evidence implementation. … Universities do not have these.” (Participant 6, academic staff)*

Meanwhile, a few participants noted that academic-practice partnership was not a “half and half” mode of partnership for the academic and clinical sides. This idea was embodied in two aspects. Firstly, the academic side needs to play a leading role in evidence synthesis, while the clinical side needs to play a leading role in evidence implementation. They do not share half and half the responsibilities in each stage of EBP. Secondly, two participants envisioned that *“With the improvement of evidence-based practice education of clinical nurses, in the future, academics may not be needed for the implementation of evidence-based practice. Although academics will still be needed to explore the theoretical and methodological topics and conduct novel research for generating evidence which could be used in future clinical implementation.” (Participant 9, Ph.D. student)*

### 3.2. Modes of Academic-Practice Partnership in EBP

Participants described that academic-practice partnership had been built through many approaches. However, most partnerships were superficial without intensive collaborations.

#### 3.2.1. Various Academic-Practice Partnerships

The academic-practice partnership in EBP could be and has been built through different forms. *“It (academic-practice partnership) has different forms. The role and responsibility of different persons may be different in different forms.” (Participant 5, academic staff)*

The academic-practice partnership in EBP mentioned by participants could be divided into different types, such as formal and informal partnerships; individual level, organizational level, and individual-organizational level collaborations; and academic nurse-dominated and clinical nurse-dominated partnerships. Seven participants mentioned the informal and individual academic-practice partnership. For example, *“During that period (of making evidence synthesis), I contacted many people, such as Ph.D. candidates and master students in a nursing school. I consulted them on many issues.” (Participant 20, clinical nurse)* Four participants mentioned the formal and organizational academic-practice partnership. For example, *“Our hospital signed with RNAO* (*Registered Nurses' Association of Ontario) to collaborate in evidence implementation. We will use their guidelines in our hospital.” (Participant 21, clinical nurse)* One participant mentioned *the* formal and individual-organizational academic-practice partnership. *“We signed a contract of one year with a Ph.D. candidate of the University of *^*∗∗∗*^* who was experienced in evidence-based practice. Based on clinical nurses' needs, he shared lectures on evidence translation with us every month.” (Participant 21, clinical nurse)* The forms of academic-practice partnership described by participants indicated that most partnerships dominated by academic nurses were used for evidence synthesis, while partnerships dominated by clinical nurses were for evidence implementation.

The partnerships between EBP organizations (such as JBI, JBI Collaboration Entity, and RNAO) and clinical nurses were the form most frequently mentioned. *“We (a JBI collaborating centre) trained many head nurses and clinical nurses in hospitals. … and we decided to use the (evidence implementation) project-based training programs for promoting the academic-practice partnership in evidence-based practice.” (Participant 2, academic staff)*

#### 3.2.2. Superficial Academic-Practice Partnerships

Most existing partnerships described by participants were superficial although they had been built through different forms in different settings and contexts. These characteristics of superficiality were reflected by scattered tasks, independent working, and a lack of mutual and intensive interaction.

Most collaborators in academic-practice partnership regarded EBP as scattered tasks without a holistic view. They worked independently and just looked for temporary collaboration, i.e., help from the other role in a specific aspect of EBP, rather than persistent collaboration throughout the whole project. As one participant shared:

*“We consulted academic staff in some steps. After we transferred the best evidence to the audit criteria in the evidence implementation project, we asked the academic staff for suggestions. They did not provide us with comments for revision. … However, after I completed the paper of this evidence implementation project and asked for academic staff to review our paper, they found problems with audit criteria, such as not being comprehensive and specific enough.” (Participant 19, clinical nurse)*

Another participant explained: *“There wasn't a good collaborative atmosphere, you do your things and I do mine.” (Participant 9, Ph.D. student)* There was a lack of interaction among collaborators. *“There were no close connections between the clinical nurses and the mentors (academic nurses who were experienced in evidence-based practice) in the training program of evidence-based practice.” (Participant 21, clinical nurse)* There are many challenges to academic-practice partnership in EBP which could explain the superficial partnerships.

### 3.3. Challenges of Academic-Practice Partnership in EBP

Participants reported multilevel challenges which hindered initiating, building, and maintaining the partnerships between nursing academics and clinicians for supporting EBP.

#### 3.3.1. Lack of Supportive Policies

Supportive policies from governments and organizations, which create a more friendly and encouraging environment for EBP and academic-practice partnership, are required for fostering, stimulating, and realizing the full potential of academic-practice partnership in EBP. These supportive policies are essential for strategic partnerships on organizational levels and intensive individual partnerships. However, participants reported that there was a lack of supportive policies from governments and organizations for EBP and academic-practice partnership.

*“There are few supportive policies on evidence-based practice in our country and hospital. Clinical nurses do not think that evidence-based practice could bring them direct benefits. … Many head nurses pay more attention to original research which could bring them more benefits than evidence-based practice. [This is] because achievements in scientific research are important indicators of many individual and organizational evaluation systems.” (Participant 16, head nurse)*

*“The policy (which only recognizes the first and corresponding authors) on achievements identification in most organizations hinder the collaboration (between academic and clinical nurses).” (Participant 1, academic staff)*

*“Many supports are just limited [to a superficial positive] attitude [towards evidence-based practice],… There is no specific support at the policy and practice level. For example, we do not have the policy to promote plans for human resources, time, and financial support to support evidence-based practice. Just saying “This is a good thing, and we need to do it.”” (Participant 5, academic staff)*

#### 3.3.2. Limited Training in EBP

Participants perceived that training was limited to equipping the academic and clinical nurses with essential evidence-based knowledge and skills. Insufficient and ineffective training on EBP could not provide qualified collaborators for academic-practice partnership in EBP.

*“In our nursing school, only two academic staff received training programs on evidence-based practice. … How can you talk more about an academic-practice partnership in the condition that there is very limited academic staff mastering the methodology of evidence-based practice? It is a real problem.” (Participant 3, academic staff)*

*“(In the ward I worked before,) most senior clinical nurses are around 40 to 50 years old. Most of them never even heard about evidence-based practice. Although most young clinical nurses in hospitals know evidence-based practice. They did not receive systematic training programs on evidence-based practice.” (Participant 7, Ph.D. student)*

*“(As a teacher of evidence-based practice,) I only accept systematic training programs on theoretical knowledge of evidence-based practice. However, I never conduct an evidence implementation in the real world. This brought me some problems in my teaching.” (Participant 3, academic staff)*

#### 3.3.3. Invisible Hierarchy between Academics and Practitioners

An invisible hierarchy between academics and practitioners was found in the interviews. In this hierarchy, academics were treated as being of a higher rank. Many participants' wording and statements reflected this subtle hierarchy although only one participant directly mentioned the exact word “hierarchy.”

*“I feel that some nursing academics sometimes are arrogant. …“Compared to clinical nurses,” they think they are more… (pause). There is a hierarchy. Although we never talk about this frankly. But it indeed exists. Clinical nurses find it difficult to collaborate with academics.” (Participant 9, Ph.D. student)*

*“Professors from universities guided us in the whole process of the project.” (Participant 22, clinical nurse)*

*“I'm a little embarrassed. Because I think the professors*^*∗∗∗*^*and*^*∗∗∗*^*in nursing school are so successful and busy. I feel embarrassed to bother them and ask too many questions.” (Participant 18, Head nurse)*

This invisible hierarchy was an unspoken truth but hindered clinical nurses from establishing partnerships with academic nurses.

#### 3.3.4. Insufficient Attention to Clinical Nursing Practice

Insufficient attention to clinical nursing practice, especially by academic nurses, hindered the academic-practice partnership in EBP. Many participants mentioned that consistency in the concerned areas is important for establishing and maintaining a partnership. *“I have the research areas I am interested in, and my energy is limited. Therefore, I cannot help other people to realize (the things they are interested in).” (Participant 1, academic staff)* However, *“Many academic staffs in nursing schools do not focus on research closely related to clinical nursing practice. This would lead to a gap between research and practice. And can we find appropriate academic staffs and students to collaborate with clinical nurses to promote evidence-based practice?” (Participant 1, academic staff)*

The lack of evidence on many clinical nursing problems also suggested that there was a lack of nursing academics focusing on clinical nursing practice. *“We often found there was no evidence which could answer our clinical questions. Therefore, in evidence implementation projects, we have deliberately ignored these clinical problems without evidence.” (Participant 22, Clinical nurse)*

#### 3.3.5. Lack of Interactive Communication

Lack of interactive communication between partners led to a lack of mutual and intensive interaction in academic-practice partnership. A common format of communication in the partnerships was “*[We] just asked and answered questions without fully understanding and discussing.”* An academic participant shared, *“I felt that they (clinical nurses) often want a specific and clear answer. When they answer questions, they often expect an answer of YES or NO.” (Participant 5, academic staff)* Conversely, a clinical nurse shared that *“Academic nurses do not have enough understanding of the issues related to our specialty. … Therefore, they did not understand some small points in nursing practice which we thought were important. We explained that to them. However, we found they did not accept and understand very well.” (Participant 16, Head nurse)*

Furthermore, the different understandings of academic nurses and clinical nurses on evidence implementation also indicated that there was a lack of interactive communication between partners. Many clinical nurses thought evidence implementation should be conducted strictly following related methodology and steps. They often use words like “right” and “wrong” in their description of their experience in evidence implementation. *“I often felt that my theoretical knowledge is limited during every phase in the evidence implementation project. … I often worried I did something wrong.” (Participant 18, Head nurse)* Three academic nurses mentioned clinical nurses were trapped by methodology in EBP. *“Evidence-based practice means the implementation of evidence. We have a set of methods. However, we should not be restricted by the methodology. Actually, it is a kind of practice model.” (Participant 8, Ph.D. student)* We found that, because of the different understandings, clinical nurses focused more on the methodology and procedures of evidence implementation projects, while academic nurses thought clinical nurses should focus more on problems in the real context rather than be limited by the methodology.

#### 3.3.6. Nonmutual Needs on Specific Steps of EBP

Academic nurses and clinical nurses had collaboration intentions and needs for the overall EBP process. However, they did not have mutual needs on specific steps. For example, clinical nurses required the assistance of academic nurses in evidence synthesis, while they did not need academic nurses during the evidence implementation in the clinical context which was indeed the part academic nurses were interested in. In contrast, academic nurses required clinical nurses to implement evidence in nursing practice, while they did not need clinical nurses during the evidence synthesis–which was indeed the part clinical nurses were often most interested in learning about. The inconsistency of needs on specific steps led to independent work on scattered tasks, which hindered the establishment and maintenance of a strong partnership.

*“I am most interested in the part of evidence implementation.” (Participant 13, Master student)*

*“To be honest, I think I do not need the help (from academic nurses) relating to clinical issues.” (Participant 16, Head nurse)*

*“For example, he/she (the clinical nurse) has gotten evidence from us. Once he/she started (the evidence implementation), he/she would not fully involve us in the process of the evidence implementation in the clinical context. …We did not need them (clinical nurses) in evidence synthesis once the PICO was confirmed.” (Participant 4, academic staff)*

#### 3.3.7. Lack of Theoretical Guidance on Collaboration

Academic-practice collaborations in EBP described by participants were limited to the practice level and often lacked a theoretical basis. The lack of theoretical guidance for academic-practice partnership in EBP made it difficult to build and sustain effective and successful cooperation.

*“I want to know how a clear pathway of collaboration deal with clinical problems. However, there is no clear pathway of collaboration (between academics and practitioners).” (Participant 10, Ph.D. student)*

*“The problem is how to do this mode (of academic-practice partnership in evidence-based practice) in detail and what is the mode in detail, this is no relevant guidance yet. … How can cooperation be more effective and feasible?” (Participant 5, academic staff)*

Notably, all participants directly or/and indirectly mentioned that there were many barriers to evidence implementation (including institutional-related barriers, interdisciplinary barriers, individual-related barriers, and evidence-related barriers). However, many barriers could not be solved only by the academic-practice partnership. *“I felt that many people were reluctant to change because we are used to staying in an environment that we are very familiar with. If the working environment still works and it doesn't bring me any trouble, why should I change it? This kind of common thinking may be a big obstacle.” (Participant 7, Ph.D. student)* These barriers (e.g., inadequate staffing, lack of institutional support, and lack of teamwork with other professionals) would also hinder the evidence implementation even under the academic-practice partnership.

### 3.4. Benefits of Academic-Practice Partnership in EBP

Still, for all of these challenges, participants believed that both academic and clinical nurses could benefit from the academic-practice partnership, and improved collaboration would influence the quantity and quality of the benefits of EBP. They listed several potential benefits: (1) improving competencies in EBP and collaboration, (2) promoting resource integration, (3) promoting EBP, and (4) bridging research-education-practice gaps.

#### 3.4.1. Improving Competencies in EBP and Collaboration

Academic-practice partnership could improve competencies in EBP and collaboration. The group-level competency in EBP of academic nurses and clinical nurses could be improved immediately, once the partnership is established. *“As a group of clinical and academic nurses, we are certainly more capable and resourceful than the two parties alone in the practice of evidence-based nursing. And they can achieve the effects of that one plus one is more than two.” (Participant 6, academic staff)* The individual-level competencies in EBP and collaboration could be improved through learning by doing and collaborative communications. *“While we were communicating with clinical teachers, they provided us with many suggestions from clinical perspectives. We had many communications, even only during the process of identifying a clinical problem, every person may have an improvement in evidence-based practice and collaboration competencies.” (Participant 7, Ph.D. student)*

#### 3.4.2. Promoting Resource Integration

Academic-practice partnership could integrate organizational resources of the academic side and clinical side required by EBP. *“When we were in the partnership, we shared the resources of our organizations.” (Participant 22, Clinical nurse) Academic-practice partnership can provide partners with more available resources, for example, academic resources of the universities such as training programs and databases, the clinical practice environment in the hospital, professionals in other disciplines, and leadership in evidence-based nursing.” (Participant 6, Academic staff)*

#### 3.4.3. Promoting EBP

Participants shared that academic-practice partnership could increase the quantity and quality of EBP. They suggested that partnerships could lead to persistence and success in implementing EBP initiatives:

*“It would be great if academic nurses collaborate with us. If you can ask for some help when you are in trouble,… I will not feel it is difficult to achieve the desired goal. … In addition, when I want to give up, the academic partners can give me a push to persist and complete the evidence-based practice project.” (Participant 19, clinical nurse) “The support from academic staff helped us to insist and then complete the project better.” (Participant 18, Head nurse)*

#### 3.4.4. Bridging Research-Education-Practice Gaps

Academic-practice partnership could bridge the gaps between research, education, and practice which are critical barriers to EBP. For bridging the research-education gap, the academic-practice partnership in EBP could promote academic and clinical teachers to provide nursing students with more knowledge based on research in nursing education. *“I found some contents in the textbooks were inconsistent with available and best evidence. … If the academic and clinical teachers could not provide nursing students with evidence-based knowledge. This would limit evidence-based practice. I think the academic-practice partnership may solve this problem.” (Participant 6, academic staff)*

For bridging the education-practice gap, the academic-practice partnership in EBP could provide nursing students with more context-related knowledge and practice experience in the education of EBP. *“When I took courses in evidence implementation for students, it was hard for me to give many examples. If I could collaborate with clinical nurses on more evidence implementations. I think the courses will be more interesting and practical.” (Participant 4, academic staff) “I only have the experience of evidence synthesis. … I went through a period of self-doubt and thought the things I did were useless. … If I could join this kind of (academic-practice partnership) group, I could go to the next steps (i.e., evidence dissemination and evidence implementation) rather than only limit myself to evidence synthesis.” (Participant 11, Ph.D. student)*

For bridging the research-practice gap, the academic-practice partnership in EBP could promote more clinical-related nursing studies to provide more applicable evidence for nursing practice. *“Co-creating knowledge can make our research more likely to be used in practice.” (Participant 9, Ph.D. student)*

## 4. Discussion

The study aimed to explore academic and clinical nurses' perceptions and experiences of academic-practice partnership in EBP. In this study, participants believed that academic-practice partnership was a necessary strategy to promote EBP and to create a win-win situation for both academic and clinical nurses and their organizations. Partnerships could be built through different modes. Nevertheless, most current academic-practice partnerships were superficial.

The complementarity of the academic side and clinical side leads to the necessity of academic-practice partnership in EBP [17, 24]. Considering clinical practice setting is the starting point and ending point of evidence (i.e., research questions and evidence implementation), clinical nurses and healthcare organizations are the essential elements in EBP. As some participants mentioned in this study, clinical nurses' need for academic nurses in EBP may decrease, with the popularization of high-quality education on EBP. However, as revealed in this study, the limited education opportunities available for clinical nurses are a big challenge that will not be solved in the short term. Similarly, other studies indicated that lack of relevant knowledge and skills has long been one of the biggest barriers for clinical nurses to conduct evidence-based practices [5, 25]. Our study findings showed that through the collaboration with academic nurses, clinical nurses could obtain immediate and complementary resources and guidance for evidence-based practice. Therefore, the academic-practice partnership would be an essential model for promoting high-quality EBP in a long time.

Different collaboration forms provide the academic-practice partnership with more possibilities for promoting EBP. However, most collaboration activities between the academic side and clinical side were superficial, which limited the effectiveness of academic-practice partnership. Close partnerships should be based on substantial, interactive, frequent, and lasting collaboration activities, which often occur in the context of strategic partnerships with organizational and leadership supports [14]. However, most collaboration activities described by participants in this study involved scattered tasks, independent working, and lacked mutual and intensive interaction.

We found that multilevel challenges hinder EBP. This finding is corroborated by previous research, which found multiple barriers to the initiation, building, and maintenance of the partnerships between the academic side and clinical side for supporting EBP. These barriers included the lack of supportive policies; limited training about EBP; invisible hierarchy between academics and clinicians; insufficient attention to clinical nursing practice; lack of interactive communication; nonmutual needs on specific steps of EBP; and lack of theoretical guidance on collaboration [16]. Multilevel strategies are required to overcome these challenges to reach close academic-practice partnerships.

Firstly, policy supports are essential for long-term collaboration in that policies are essential for cultivating innovation-encouraging and collaboration-friendly environments which is the basis for close partnerships to promote EBP [26, 27]. Particularly, governments, academic, and clinical institutions should develop policies that integrate EBP-oriented and academic-practice partnership-oriented requirements and performance appraisal on staff and working teams. In addition to providing a supportive atmosphere, policies could also strongly push institutions and individuals to take measures to overcome other barriers to academic-practice partnership in EBP.

Secondly, individual education on EBP provides the basis for academic-practice partnership in EBP. Collaboration on activities of EBP requires academic nurses and clinical nurses to be equipped with basic knowledge and skills provided by effective EBP courses and training programs. However, the quantity and quality of education on EBP are still limited [28]. To present more EBP education opportunities, we should cultivate more academic and clinical staff to be equipped with the necessary competencies to teach EBP [5]. Meanwhile, we should include EBP into the curriculum at all stages of nursing professional higher education (i.e., bachelor, master, and Ph.D.) [5, 29, 30]. Registered Nurses'. A systematic review [5] proposed several suggestions to improve the effectiveness of teaching EBP: using the educational strategy of combination and married with clinical exposures; adequately contributing to the student nurses' acquisition of EBP knowledge and implementation; using theoretical frameworks and models, interactive teaching styles, and appropriately sequencing duration, timing, and content for teaching EBP within the curriculum; and using academic-practice partnerships for teaching EBP, especially in the resource-constrained settings.

We also found that an invisible hierarchy between academic and clinician nurses limits deep collaborations because real partnerships cannot be built when there is imbalanced power-sharing between the academic and the clinical staff [14]. In this study, the relationship between the two sides was more like guidance by academics to clinicians, rather than partnership. Other scholars have found that insufficient attention to clinical nursing practice leads to a lack of motivation for EBP and few academic nurses focusing on research related to clinical nursing practice, which hinders the academic-practice partnership in EBP [4]. To deal with these two challenges (i.e., invisible hierarchy and insufficient attention to clinical nursing practice), the emphasis on “high-quality practice-oriented” should replace the emphasis on “research-oriented” in policies and performance appraisal systems to reduce the invisible hierarchy between academics and practitioners, increase the attention of academics on clinical nursing practice, and promote “interactive communication” rather than “consultation conversations” between academics and practitioners. Furthermore, the lack of interactive communication in the academic-practice partnership may be also because there was an “academic-practice gap” [31]. In other words, academic nurses and clinical nurses have different professional experiences and training systems, leading to gaps in theoretical knowledge, clinical experience, and ineffective communication between academic and clinical nurses. Therefore, compatible and understandable languages to achieve shared understanding for both sides, voluntary and frequent communications, deeper understanding of the context, and experience of the other side are crucial to overcoming the academic-practice gap for promoting interactive communication [31].

Finally, there is a lack of specific theoretical guidance on academic-practice collaborations in the context of EBP although there are models, frameworks, and principles guiding EBP and academic-practice partnerships, respectively [14, 32, 33]. Meanwhile, although there is theoretical guidance (i.e., academic-practice partnership logic model and AACN's guiding principles for academic-practice partnerships) proving implications for academic-practice partnerships on the organizational level, there is a lack of theoretical guidance on individual collaborations [14, 33]. Considering individual-level collaborations are the prerequisite and core elements of organizational-level partnerships, the specific and theoretical research guiding the organizational-level partnerships and individual-level collaborations are both necessary to promote effective academic-practice partnership in EBP. It is noted that, excepting dealing with the above challenges of academic-practice partnerships, the measures of other barriers to EBP are also crucial for promoting academic-practice partnership in EBP [4, 7].

This study suggested that successful academic-practice partnership could improve competencies in EBP and collaboration, promote resource integration, EBP, and bridge research-education-practice gaps. The quality and degree of academic-practice partnership would influence the quantity and quality of the benefits, which were supported by other studies [11, 16]. However, existing evidence on approaches to develop academic-practice partnership is limited. More high-quality studies with rigorous research designs (e.g., experimental trials) and applying multimethods (e.g., mixed-method studies) are required to evaluate the short-term and long-term outcomes effectively and comprehensively [13, 16].

## 5. Limitations

There are three main limitations in this study. Firstly, most of the participants (i.e., 19 participants) are from central China (which is at a median level on economic and healthcare service levels); other regions with different contexts might have different modes of collaboration. Secondly, participants having the experience of deep collaboration and close partnership in this study were limited because the academic-practice partnership in EBP was limited in the context of this study. The participants with rich experience in close partnerships may provide richer or even different information on academic-practice partnership in EBP. Thirdly, the experience of academic-practice partnership in EBP of participants was in the past. The recall difficulty could also limit the information's accuracy and adequacy.

In the future, other qualitative data collections (e.g., observational method and timely interviews) during the process of academic-practice partnership in EBP are important, which could provide more exact and vivid examples of views and experiences on academic-practice partnership in EBP.

## 6. Conclusions

Academic-practice partnership is a win-win strategy for both the academic side and clinical side to promote EBP. Different modes of academic-practice partnership provide academic and clinical nurses with more possibilities and better chances to promote the EBP. However, challenges in academic-practice partnership in evidence-based nursing require multilevel measures to provide a better environment to initiate, build, and maintain intensive collaborations between academic and clinical nurses.

### 6.1. Implications for the Profession and/or Patient Care

The collaborations between academic and clinical nurses are the basis and core of academic-practice partnerships. The individual-level collaborations on EBP activities could improve EBP competencies, resources, and education, to promote EBP and the professional development of academic and clinical nurses. Meanwhile, to effectively initiate, build, and maintain close collaborations on the individual level, leaders in academic and clinical organizations should make efforts (e.g., providing supporting policies, signing a memorandum of cooperation, providing time, resources, and incentive system) to cultivate a friendly environment for academic-practice partnership in EBP.

## Figures and Tables

**Table 1:** Basic characteristics of participants (*n* = 22).

	Mean ± SD, range
Age (years)	31.14 ± 6.39, (22–48)
Evidence-based Nursing Practice Competence Score^a^	74.36 ± 6.46, (65–92)

	*n* (%)

Gender	
Female	28 (81.82%)
Male	4 (18.18%)
Educational degree	
Baccalaureate	5 (22.73%)
Master	10 (45.45%)
Ph.D.	7 (31.82%)
Position	
Academic staff	6 (27.27%)
Master's student (professional degree)	2 (9.09%)
Master's student (academic degree)	3 (13.64%)
Doctoral candidate	4 (18.18%)
Head nurse	3 (13.64%)
Bedside nurse	4 (18.18%)
Experience in EBP projects	
Yes	19 (86.36%)
No	3 (13.64%)
Experience in academic-practice partnership in evidence-based practice	
Yes	14 (63.64%)
No	8 (36.36%)

*Note.* ^a^Total score on the evidence-based nursing practice competence scale ranges from 0 to 92. This Chinese measurement was developed and validated by Wang et al. in 2017 [21]. More details of this scale can be found in the note of Supplementary Material Table S1.

**Table 2:** Themes and subthemes generated in this study.

Themes	Subthemes
(1) Necessities of academic-practice partnership in promoting EBP	(1.1) Complementary competencies for partnership
	(1.2) Complementary resources for partnership

(2) Modes of academic-practice partnership in EBP	(2.1) Various academic-practice partnerships
	(2.2) Superficial academic-practice partnerships

(3) Challenges of academic-practice partnership in EBP	(3.1) Lack of supportive policies
	(3.2) Limited training in EBP
	(3.3) Invisible hierarchy between academics and clinicians
	(3.4) Insufficient attention to clinical nursing practice
	(3.5) Lack of interactive communication
	(3.6) Nonmutual needs on specific steps of EBP
	(3.7) Lack of theoretical guidance on collaboration

(4) Benefits of academic-practice partnership in EBP	(4.1) Improving competencies in EBP and collaboration
	(4.2) Promoting resource integration
	(4.3) Promoting EBP
	(4.4) Bridging research-education-practice gaps

## Data Availability

The data that support the findings of this study are available upon request from the corresponding author. The data are not publicly available due to their containing information that could compromise the privacy of research participants.

## Supplementary Materials

Supplementary Materials Table S1: general information of participants at the time of interview.
