# Supplementary material for: Academic and Clinical Nurses' Perceptions and Experiences on Academic-Practice Partnership in Evidence-Based Practice: An Interpretive Description
Source: J Nurs Manag. 2023 Sep 19;2023:2955731. doi: 10.1155/2023/2955731 (PMC11919230; doi:10.1155/2023/2955731)
Supplement: Supplementary Materials — Table S1: general information of participants at the time of interview. [file 2955731.f1.docx]

**Table S1. General information of participants at time of interview (n=22)**

| **Participants** | **Age** | **Gender** | **Educational degree** | **Position** | **Score of Evidence-Based Nursing Practice Competence Scale ^a^** | **Experience in EBP projects** | **Experience in academic-practice partnership in EBP** |
| --- | --- | --- | --- | --- | --- | --- | --- |
| Participant 1 | 34 | Female | Ph.D. | Academic staff in university nursing department | 66 | Evidence synthesis | No |
| Participant 2 | 48 | Female | Ph.D. | Academic staff in university nursing department | 92 | Evidence synthesis, Evidence dissemination,  Evidence implementation | Yes |
| Participant 3 | 34 | Male | Ph.D. | Academic staff in university nursing department | 82 | Evidence synthesis | No |
| Participant 4 | 32 | Female | Ph.D. | Academic staff in university nursing department | 82 | Evidence synthesis,  Evidence implementation | Yes |
| Participant 5 | 33 | Male | Ph.D. | Academic staff in university nursing department | 77 | Evidence synthesis,  Evidence implementation | Yes |
| Participant 6 | 30 | Female | Ph.D. | Academic staff in university nursing department | 78 | Evidence synthesis, Evidence implementation | Yes |
| Participant | 28 | Female | Master | Ph.D. student in university nursing department | 71 | Evidence synthesis | No |
| Participant 8 | 27 | Female | Master | Ph.D. student in university nursing department | 68 | Evidence synthesis | No |
| Participant 9 | 29 | Male | Master | Ph.D. student in university nursing department | 70 | Evidence synthesis, Evidence implementation | Yes |
| Participant 10 | 28 | Male | Master | Ph.D. student in university nursing department | 70 | Evidence synthesis, Evidence implementation | Yes |
| Participant 11 | 27 | Female | Master | Ph.D. student in university nursing department | 80 | Evidence synthesis | Yes |
| Participant 12 | 22 | Female | Baccalaureate | Master student (professional degree) in university nursing department | 65 | Evidence synthesis | No |
| Participant 13 | 23 | Female | Baccalaureate | Master student (academic degree) in university nursing department | 72 | Evidence synthesis | No |
| Participant 1 | 22 | Female | Baccalaureate | Master student (academic degree) in university nursing department | 80 | Evidence synthesis | No |
| Participant 15 | 23 | Female | Baccalaureate | Master student (professional degree) in university nursing department | 73 | Evidence synthesis | No |
| Participant 16 | 37 | Female | Master | Head nurse in hospital | 73 | Evidence synthesis, Evidence dissemination, Evidence implementation | Yes |
| Participant 17 | 37 | Female | Ph.D. | Head nurse in hospital | 69 | Evidence synthesis, Evidence dissemination, Evidence implementation | Yes |
| Participant 18 | 39 | Female | Master | Head nurse in hospital | 69 | Evidence dissemination, Evidence implementation | Yes |
| Participant 19 | 36 | Female | Master | Clinical nurse in hospital | 69 | Evidence dissemination, Evidence implementation | Yes |
| Participant 20 | 30 | Female | Master | Clinical nurse in hospital | 78 | Evidence synthesis, Evidence dissemination, Evidence implementation | Yes |
| Participant 21 | 29 | Female | Master | Clinical nurse in hospital | 77 | Evidence synthesis, Evidence dissemination, Evidence implementation | Yes |
| Participant 22 | 37 | Female | Master | Clinical nurse in hospital | 75 | Evidence dissemination, Evidence implementation | Yes |

Note:

^a^ Total score of Evidence-Based Nursing Practice Competence Scale ranges from 0 to 92. The Evidence-based Nursing Practice Competence Scale (EBNPCS) was used to measure the EBP competence of nursing personnel with the EBP training experience. This Chinese measurement was developed and validated by Wang et al in 2017 (Wang *et al.* 2017) . It could be used for different types of nursing personnel. EBNPCS includes 23 items rated on a five-point Likert scale (i.e., 0 score - “know nothing about this part”, 4 scores - “completely agree”) in four subscales, i.e., Evidence Search and Evaluation Competence (1-7 items), Evidence Synthesis Competence (8-12 items), Evidence Dissemination Competence (13-15 items), Evidence Implementation Competence (16-23 items). A higher score suggests that the participant is equipped with better EBP competence. The scale-level content validity index (S-CVI) was 0.987. The *Cronbach’s α* for the scale and subscales was 0.951, 0.873, 0.916, 0.902, 0.855, respectively. The test-retest reliability was 0.900.

**Reference:** Wang Y., Hu Y., Zhou Y. & Xing W. (2017): Development and validation of evidence-based nursing practice scale. Journal of Nursing Science 32, 49-52+59. In Chinese.
